# *Chlamydia trachomatis* Infection Control Programs: Lessons Learned and Implications for Vaccine Development

**DOI:** 10.1155/2011/754060

**Published:** 2011-11-14

**Authors:** Jean M. Chavez, Rodolfo D. Vicetti Miguel, Thomas L. Cherpes

**Affiliations:** Department of Pediatrics, University of Pittsburgh School of Medicine, Rangos Research Center, Room 9123, 4401 Penn Avenue, Pittsburgh, PA 15224, USA

## Abstract

*Chlamydia trachomatis* control efforts that enhance detection and treatment of infected women may paradoxically increase susceptibility of the population to infection. Conversely, these surveillance programs lower incidences of adverse sequelae elicited by genital tract infection (e.g., pelvic inflammatory disease and ectopic pregnancy), suggesting enhanced identification and eradication of *C. trachomatis* simultaneously reduces pathogen-induced upper genital tract damage and abrogates formation of protective immune responses. In this paper, we detail findings from *C. trachomatis* infection control programs that increase our understanding of chlamydial immunoepidemiology and discuss their implications for prophylactic vaccine design.

## 1. Introduction

An estimated 90 million individuals sexually acquire *Chlamydia trachomatis *infection each year (including 45 million in Asia, 15 million in Africa, and 4 million in the United States [1]), and in all these geographic regions the highest prevalences of chlamydial genital tract infection are found among adolescents and young adults [2]. Although *C. trachomatis* is a common cause of male nongonococcal urethritis [3], female genital tract infections represent more significant threats to reproductive health. Morbidities associated with *C. trachomatis* genital tract infections in women include pelvic inflammatory disease (PID) and its sequelae of chronic pelvic pain, ectopic pregnancy, and tubal infertility [4]. 

## 2. Shortcomings and Benefits of Infection Control Programs

During the past 25 years, many areas in Europe and North America implemented infection control programs to reduce sexual transmission of *C. trachomatis* [5]. These programs typically relied upon widespread screening and prompt treatment of asymptomatic individuals as a conduit for decreased population infectivity [6–8]. Applying these principles, one regional United States program reduced *C. trachomatis* prevalence 60% among young women during the first 9 years of its existence [9]. However, similar screening and treatment of young women in this region during the succeeding 7 years was associated with a 46% increase in chlamydial positivity [10]. This scenario was repeated in British Columbia—after introduction of a *C. trachomatis* infection control program case rates fell from 216 to 104 cases per 100 000 individuals but then steadily climbed to 193 cases per 100 000 individuals [11]. In fact, nearly all countries implementing large-scale chlamydial control programs have reported increased case report numbers despite ongoing control efforts [12–14], suggesting expanded earlier treatment may enhance population susceptibility to *C. trachomatis* infection. Although the higher prevalences of *C. trachomatis* seen in areas with active surveillance may have been sequelae to increased screening of higher risk women or increased use of more sensitive diagnostic tests, at least in 1 such surveillance program higher prevalence appeared to reflect actual increases in chlamydial positivity [15]. Providing stronger support for the supposition that *C. trachomatis* infection control programs increase population susceptibility to infection, British Columbia saw reinfection rates rise from 9.7 to 53.2 cases per 100,000 individuals in the midst of widespread control efforts [11, 15]. 

Concurrent with higher incidences of reinfection, however, *C. trachomatis* control programs have reduced genital tract complications elicited by infection. For example, concomitant with steady increases in *C. trachomatis* case numbers, health officials in San Francisco County, Calif, observed dramatic decreases in PID and ectopic pregnancy cases [16]. Earlier diagnosis and treatment of genital tract chlamydial infections was also associated with sharp reductions in ectopic pregnancy rates in Norway and Sweden [17, 18]. Despite the substantially increased rates of *C. trachomatis *infection documented in British Columbia [11], surveillance data demonstrated robust decreases in annual case numbers and rates of PID, ectopic pregnancy, and tubal factor infertility [19]. Taken together, these data imply that enhanced detection and earlier treatment of infected women achieved upon implementation of *C. trachomatis* infection control programs may have been responsible for reduced incidences of the adverse outcomes associated with ascension of this pathogen into the upper genital tract. These data further imply that persistent *C. trachomatis* infection, not simply acquisition or reinfection, may be the scenario most likely responsible for development of PID, ectopic pregnancy, and tubal factor infertility. 

## 3. *C. trachomatis* Control Programs and the Arrested Immunity Hypothesis

Seminal investigations performed in British Columbia allowed Brunham et al. to first posit that *C. trachomatis* infection control programs increase population susceptibility to reinfection [11]. Their “altered immunity” hypothesis states that development of protective immune responses against *C. trachomatis* is abrogated by earlier detection and treatment of infected individuals and further argues this interrupted development of protective immunity increases the likelihood of reinfection. Evidence supporting this proposed linkage between expanded earlier treatment and increased population susceptibility to infection has been provided by both experimental and clinical investigations. Compared to untreated mice, humoral immune responses were impaired in the vaginas of mice administered doxycycline within the first 10 days of primary intravaginal chlamydial infection. Moreover, these same antibiotic-treated mice were also less protected from chlamydial reinfection [20]. Taken together, these data suggest that accelerated eradication of chlamydia from the genital tract that was mediated by doxycycline therapy may have hampered the development of protective immune responses. 

The durability of *C. trachomatis* infection among many women not receiving antichlamydial antibiotics implies protracted courses of infection may be needed for development of sterilizing immunity, while providing further support for the validity of the altered immunity hypothesis. For example, an annual clearance rate of 45% among asymptomatic Dutch women not receiving antimicrobial therapy suggested sterilizing immunity is often not achieved during the first year of a chlamydial genital tract infection [21]. A similar rate of clearance was seen in Colombia where 54% (44/82) of women not receiving antichlamydial antibiotics cleared asymptomatic genital tract infection during the first year after initial diagnosis; however *C. trachomatis* infection persisted in only 6% of this cohort after 4 years of followup [22]. These results indicate the development of sterilizing immunity against genital tract chlamydial infection is most often measured in months or years, while long-term presence of the organism in the absence of overt inflammatory symptoms highlights the highly successful parasitic relationship *C. trachomatis* has achieved with its human hosts. In addition to these natural history studies, clinical data in support of the altered immune hypothesis was generated upon completion of a C*. trachomatis* seroprevalence study enrolling 8 000 pregnant Finnish women. During the same period of time in this country in which dramatic increases in the frequency of genital tract chlamydial infection were observed [23], this study reported a 51% decrease in the prevalence of positive serum IgG antibody titers against *C. trachomatis* major outer membrane protein (MOMP) among women less than 23 years of age and a 65% decrease in positive titers among 23–28-year-old women [24]. These findings may indicate that in some women chlamydial MOMP-specific humoral responses are transitory or, conversely, that humoral responses against *C. trachomatis* are slow to develop and that earlier identification and treatment of infection impeded the development of humoral immunity. Although more speculative, it is also possible that decreasing seroprevalence of *C. trachomatis* in Finland contributed to increased population susceptibility to reinfection. This latter clinical scenario is consistent with experimental data demonstrating that chlamydial-specific antibodies were integral for protection of female mice from genital tract reinfection [25]. Further clinical investigation, however, will be needed to determine the strength of the associations between enhanced detection and treatment, impaired humoral immune responses, and increases in susceptibility to *C. trachomatis *genital tract reinfection. 

## 4. Implications for *C. trachomatis* Vaccine Development

Widespread *C. trachomatis* infection control programs reduce incidences of PID and its adverse sequelae [19, 26], but are associated with increased population susceptibility to infection. These seemingly contradictory observations interestingly help illuminate the immunoepidemiology of *C. trachomatis* infection. Although some conclusions that we draw from these epidemiological investigations remain conjectural, increased population susceptibility to chlamydial infection seen concomitantly with decreases in genital tract complications of infection indicates the following: (1) this obligate intracellular pathogen is weakly antigenic; (2) the organism requires persistent infection to elicit upper genital tract damage; (3) primary infection is associated with host immune responses that are suboptimal for immediate pathogen clearance but unlikely to damage vital upper genital tract architecture and/or this organism employs immunoevasion strategies that promote establishment of asymptomatic but persistent infection. 

Consistent with the notion of low antigenicity, *C. trachomatis* infections of the female genital tract often remain asymptomatic. Cell walls of *C. trachomatis*, like other Gram-negative bacteria, contain lipopolysaccharide (LPS), a molecule that stimulates multiple responses in infected tissue including increased secretion of pro-inflammatory cytokines, macrophage activation, and increased expression of endothelial leukocyte adhesion molecules. Ex vivo assays show that LPS is primarily responsible for the increased production of tumor necrosis factor (TNF) elicited by *C. trachomatis* elementary bodies, even though chlamydia LPS is 100-fold less potent for the production of this key pro-inflammatory cytokine than the LPS isolated from *Neisseria *species [27]. Therefore, epidemiological data suggesting that the presence of persistent genital tract infection promotes chlamydial disease expression is consistent with this ability of *C. trachomatis* to elicit less robust inflammatory responses [28]. In other words, pelvic inflammatory disease, fallopian tube scarring, and tubal infertility may more frequently result from the presence of chronic, albeit mild inflammation. Although persistent infection may be responsible for increased chlamydial disease expression, the specific immune responses that evoke this upper genital tract damage remain unknown. Mouse and nonhuman primate studies suggest IFN-*γ*-producing CD4^+^ T cells are needed for clearance of chlamydial infections, and the generation of appropriate type 1 immunity is often considered to be an integral component of any vaccine conferring protection against chlamydial disease expression [29]. On the other hand, increased IFN-*γ* production, in conjunction with other type 1 responses, may promote immunopathological responses and increase the likelihood of fallopian tube scarring and tubal factor infertility. Since genital tract *C. trachomatis* infections in nature are weakly antigenic, it is at least a theoretical concern that chlamydial vaccines that confer protection via generation of robust, more durable memory T cell responses could also elicit immunopathological damage if repetitive bouts of inflammation were elicited upon subsequent exposures to the organism. 

*C. trachomatis* is known to have evolved exclusively as a human pathogen and fact which may explain why infection usually occurs in the absence of overt inflammation. Strategies developed by this intracellular pathogen to avoid host detection or clearance include replication within membrane-bound inclusions [30], suppression of class I and II major histocompatibility complex molecule expression by infected cells [31], and its ability to capture indole to escape IFN-*γ*-mediated tryptophan starvation [32]. A high-frequency asymptomatic *C. trachomatis* infection, on the other hand, may be the consequence of host responses that evolved to minimize collateral damage to delicate upper genital tract structures. In support of this hypothesis, we recently saw that *C. trachomatis* genital tract infection was associated with increased numbers of endometrial CD4^+^ T cells, B cells, and plasma cells [33], suggesting there is a predilection for *C. trachomatis* infection to polarize endometrial inflammation toward type 2 immunity. Although type 1 immune responses are capable of clearing *C. trachomatis*, type 2 responses may have been selected as a safer alternative to more damaging type 1 responses in the upper genital tract [34]. Whether type 2 endometrial inflammation is associated with enhanced or impaired chlamydial clearance or higher or lower chances of immunopathological tissue damage remains unknown, but resolution of these uncertainties is essential for proper vaccine development. The reported linkage between enhanced early detection, decreased chlamydia-specific antibody prevalence, and increased chlamydial reinfection rates does not establish causality between impaired humoral immune responses and increased susceptibility to infection, but does provide the impetus for more complete understanding of the immune responses that may impact chlamydial vaccine efficacy. 

**Figure 1:**
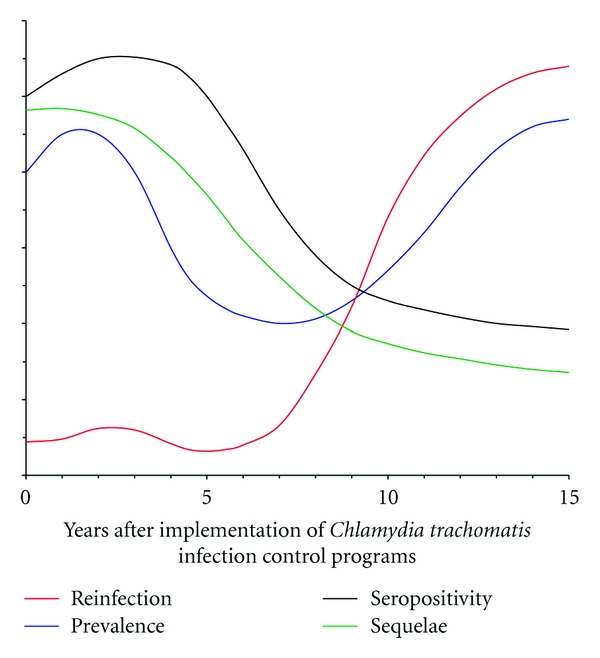
Schema summarizing outcomes associated with *Chlamydia trachomatis *genital tract infection control programs. These outcomes include increased number of chlamydial cases, increased rates of chlamydial reinfection, decreased detection of chlamydial-specific serum antibodies, and decreased rates of pelvic inflammatory disease, ectopic pregnancy, and tubal factor infertility. Although no causality between these observed outcomes has been established, chlamydial vaccine development will require better delineation of the linkage between enhanced early treatment and diminished antichlamydial humoral immunity, increased susceptibility to infection, and lower incidences of adverse reproductive tract sequelae.

In conclusion, this brief paper summarized the findings from *C. trachomatis* infection control programs that alter our understanding of the immunoepidemiology of chlamydial genital tract infection (Figure 1). Although observations from these programs suggest increased duration of infection is a risk factor for the development of PID, understanding of the specific host genetic variations and immune responses that promote genital tract damage awaits further investigation. Additional work is also needed to better inform chlamydial vaccine development, as more comprehensive understanding of the immune responses that protect against *C. trachomatis* acquisition and reinfection and prevent or elicit PID development must be achieved before clinical vaccination trials can be safely initiated.
